# Human Ovarian Tumor Cells Escape γδ T Cell Recognition Partly by Down Regulating Surface Expression of MICA and Limiting Cell Cycle Related Molecules

**DOI:** 10.1371/journal.pone.0023348

**Published:** 2011-09-14

**Authors:** Jingwei Lu, Reeva Aggarwal, Suman Kanji, Manjusri Das, Matthew Joseph, Vincent Pompili, Hiranmoy Das

**Affiliations:** 1 Cardiovascular Medicine, The Dorothy M. Davis Heart and Lung Research Institute, The Ohio State University, Columbus, Ohio, United States of America; 2 Innate Immunity, Comprehensive Cancer Center, Richard J. Solove Research Institute, Arthur G. James Cancer Hospital, The Ohio State University, Columbus, Ohio, United States of America; The University of Kansas Medical Center, United States of America

## Abstract

**Background:**

Mechanisms of human Vγ2Vδ2 T cell-mediated tumor immunity have yet to be fully elucidated.

**Methods and Findings:**

At least some tumor cell recognition is mediated by NKG2D-MICA interactions. Herein, by using MTT assay and PI-BrdU co-staining and Western-blot, we show that these Vγ2Vδ2 T cells can limit the proliferation of ovarian tumor cells by down regulation of apoptosis and cell cycle related molecules in tumor cells. Cell-to-cell contact is critical. γδ T cell-resistant, but not susceptible ovarian tumor cells escape γδ T cell-mediated immune recognition by up-regulating pErk1/2, thereby decreasing surface MICA levels. Erk1/2 inhibitor pretreatment or incubation prevents this MICA decrease, while up-regulating key cell cycle related molecules such as CDK2, CDK4 and Cyclin D1, as well as apoptosis related molecules making resistant tumor cells now vulnerable to γδ T cell-mediated lysis.

**Conclusion:**

These findings demonstrate novel effects of γδT cells on ovarian tumor cells.

## Introduction

Human gamma delta (γδ) T cells represent a small subset of T cell population that possesses distinct T cell receptor (TCR) on their surface. In contrast to approximately 50 Vα and 50 Vβ TCR gene segments that can pair to form several thousand receptor combinations in αβ T cells, there are only 6 Vγ and 4 major Vδ gene segments used by human γδ T cells [1]. Among these γδ T cell gene pairs, the Vγ2Vδ2 TCR pair is expressed on 50–75% of human peripheral blood gd T cell and thus comprise 2–5% of adult human peripheral blood CD3+ cells [2]. Vγ2Vδ2 T cell numbers in human peripheral blood can increase 2- to 10-fold (8–60% of CD3+ T cells) in a variety of infectious diseases [3]. Vγ2Vδ2 T cells may be considered part of the adaptive immune system as they have a memory phenotype, junctionally diverse TCR's that require gene rearrangement for their cell surface expression, and the ability to undergo either anergy or expansion depending on the availability of co-stimulation [4]. On the other hand, Vγ2Vδ2 T cells are also considered a part of the innate immune response. Pattern recognition by the Vγ2Vδ2 TCR allows the expansion of memory γδ T cells into a large numbers in normal adults during microbial infections [3]. These large numbers of memory T cells are capable of responding to antigens produced by microbes and thus may serve bridge the gap between the innate and adaptive immune responses [4], [5].

In developed countries, ovarian tumor is the second most common gynecological malignancy followed by endometrial tumor, but accounts for more mortality than all the remaining gynecological tumors combined [6]. Most ovarian tumor patients are diagnosed at advanced stages, and poor five-year survival was as low as 46 percent [7]. Recently, there has been a significant improvement in survival with use of immunotherapy. Sipuleucel-T, a therapeutic peripheral mononuclear cells (MNC), was approved by US Food and Drug Administration (FDA) on April 2010, as the first treatment to be able to prolonging the life of patients with advanced prostate tumor [8]. T cell infiltration has been found in many patients diagnosed with ovarian tumor, and such infiltration is significantly correlated with the five-year overall survival rate: 38.0% among patients whose tumors contained T cells and only 4.5% among patients whose tumors contained no T cells [9]. Study has shown that γδ T cells are correlated with a brief disease-free interval (p = 0.036) in advanced ovarian carcinomas [10]. This suggested an important role for γδ T cells in ovarian tumor remission and for possible therapeutic application. For this reason it is of critical importance to elucidate the mechanisms of γδ T cell interaction with ovarian tumor cells, and to understand the evasion mechanisms by which tumors escape from immunosurveillance by cytotoxic T cells.

MHC class I chain related molecules A and B (MICA and MICB, danger signals), which are widely expressed in epithelial tumor cells, and virally or bacterially infected cells, can be recognized by γδ T cells and NK cells via NKG2D; a signaling pathway is responsible for enhanced cytotoxicity against infected cells or tumors [11], [12], [13]. MICA is preserved in most mammals except for rodents and is present at high levels in gastrointestinal epithelium, and on tumor cells of epithelial origin [14]. Other ligands for NKG2D such as UL16 binding protein 1,2,3,4 (ULBP1, 2, 3, 4) RAET1G (ULBP5) and RAET1L (ULBP6) have also been reported as targets of the host immune response [15].

The expression and modulation of MICA on tumor cells have effects on cell survival [16], [17], [18]. Largely unknown, however is the mechanism by which γδ T cells affect tumor cells other than cytotoxicity and Th-1 type cytokine. In the present study, we assessed the effect of γδ T cells on proliferation of tumor cells using two ovarian tumor cell lines, A2780 and OV4, resistant and susceptible to cytotoxic lysis, respectively. We further evaluated the mechanisms by which tumor cells escape immune recognition by γδ T cells, focusing on surface molecules and cell cycle related molecules. Herein we show that the tumor cell survival from the recognition of γδ T cells is correlated with the down regulation of cell surface MICA levels and reduced expression of cell cycle related molecules, as well as up-regulation of Erk1/2 signaling. Erk1/2 inhibitor was able to bring back surface expression of MICA, induced higher levels of cell cycle related molecules and resulted in effective cytotoxic lysis by γδ T cells. These new findings help define novel mechanisms for tumor evasion against innate host immune response.

## Results

### In Vitro γδ T Cell Expansion and Characterization

As previously reported [19], [20] human γδ T cells were expanded from freshly isolated PBMCs. One million of PBMC was seeded in a well of a 24-well plate and 10 µm of risedronate was added to the culture in a complete RPMI-1640 media. Recombinant IL-2 (0.5 nM) was supplemented to the culture on day 3 and 7 during the culture. Cells were subjected to split upon overgrowth. At day 14 flowcytometric analysis was performed to assess cell expansion and to determine their phenotype. Flowcytometric analysis revealed that almost all expanded cells were CD3 positive. Among expanded cells almost 90% cells were Vδ2 positive γδ T cells (Fig. S1). There were almost no Vδ1 positive γδ T cells. Similar cells were used for the subsequent experiments.

### γδ T Cell-Mediated Apoptosis of Tumor Cells

**Figure 1:**
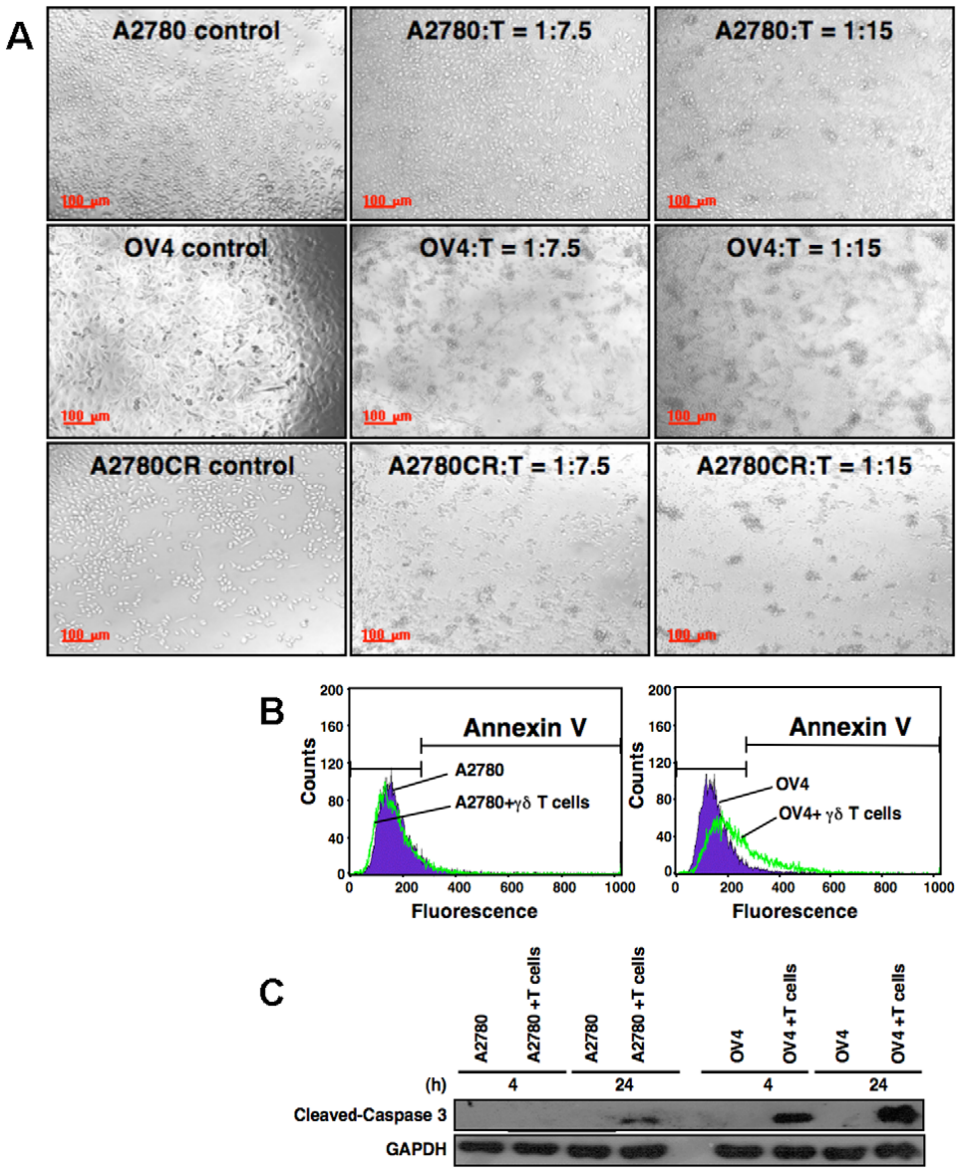
Tumor apoptosis mediated by γδ T cells. ***A.*** Morphology of tumor cells after co-culture with γδ T cells. Various ovarian tumor cell lines stated (A2780, OV4 and A2780CR) were co-cultured with γδ T cells for 24 hours with a ratio of Cancer∶T = 1∶0, 1∶7.5 or 1∶15 and morphology of tumor cells after co-culture with γδ T cells were captured by phase contrast micrographic images. Detached tumor cells formed clumps along with γδ T cells. Five experiments were performed using expanded γδ T cells isolated from three different donors. Results were similar and representative data is shown here. **B.** Flowcytometric analyses for apoptosis marker, annexin V, were performed after co-culture of tumor cells (A2780 or OV4) with γδ T cells (1∶10 ratio) for 24 h. The expression of annexin V was much higher in OV4 cells compared to A2780 cells, indicating enhanced apoptosis in OV4 cells. **C.** Higher cell death in OV4 line was also correlated with the higher level of cleaved caspase 3 protein evaluated by Western blot analysis.

As γδ T cells have the inherent characteristics of innate immunity and provide first line of defense against multiple infections and diseases, we first investigated the innate response of γδ T cells against ovarian tumor cell lines (A2780, OV4 and A2780 CR). After co-culture of tumor cells with γδ T cells, ovarian tumor cell lines showed various morphological changes (Fig. 1A). Of all those cells, OV4 cell lines showed the strongest sensitivity towards detachment, clump formation and possible death compare to all cell lines tested. Cell clusters were formed by co-culture were apparent as low as C∶T = 1∶7.5 (C = tumor cells, T = γδ T cells), which indicated that tumor cells were being killed by γδ T cells. A2780 cell line did not show much cluster formation until C∶T = 1∶15. We have selected A2780 and OV4 cell lines as representatives for resistant and susceptible respectively for γδ T cell-mediated lyses for further studies.

To further address the mediator of tumor cell death due to co-culture with γδ T cells apoptosis specific annexin V staining and flowcytometric analyses were performed. Annexin-V staining (which binds with the phosphatidylserine expressed on the apoptotic cells) data revealed that OV4 cells were more susceptible for apoptosis compared to A2780 cells. After exposure of tumor cells to γδ T cells at a ratio of 1∶10 there were no apparent changes in the expression of phosphatidylserine in A2780 cells. However, over 30% cells were apoptotic in OV4 cell line (Fig. 1B). This difference in apoptosis between the two cell lines is further confirmed by protein analyses. It was reported that FasL and perforin/granzyme B are two common pathways used by cytotoxic lymphocytes to induce apoptosis on target cells. Both pathways converge at caspase-3 pathways through different mechanisms [21], [22]. Thus, we used cleaved caspase 3 as a marker for evaluating apoptosis induced by γδ T cells on tumor cells. Cleaved caspase 3 was detectable as early as 4 h point in OV4 cell line, where as it was not detected at the same time point in A2780 cell line (Fig. 1C). Even though cleaved caspase 3 was detectable after 24 h in A2780 cells, level of expression was remarkably low compare to OV4 cell line (Fig. 1C). This result is consistent with the cluster formation, which is higher in OV4 cells.

### Inhibition of Tumor Cell Proliferation

**Figure 2:**
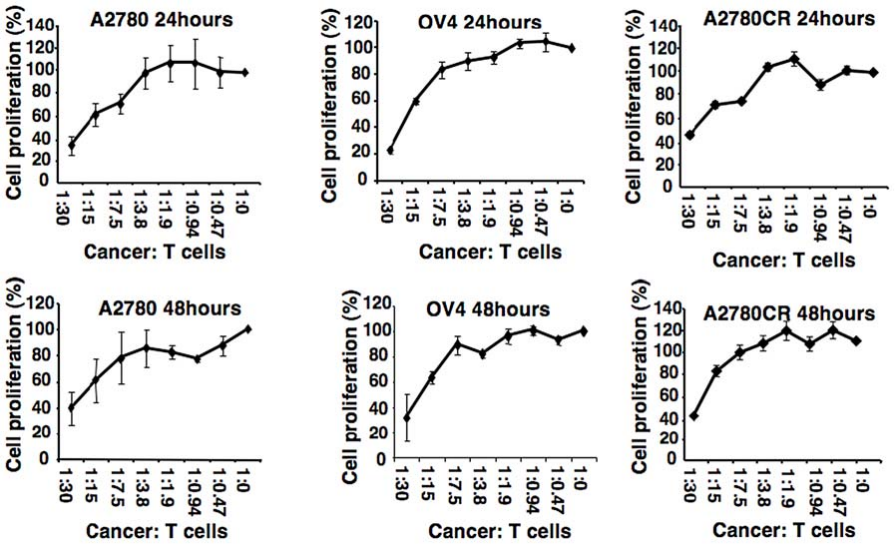
Proliferation of tumor cells in presence of γδ T cells. Ovarian tumor cell lines (A2780, OV4 and A2780CR) were co-cultured with various ratios of γδ T cells for 24 and 48 hours. MTT assay was performed to evaluate cell proliferation after gentle removal of γδ T cells after 24 and 48 hours of co-culture.

As γδ T cells have the inherent characteristics of innate immunity and provide first line of defense against multiple infections and diseases, we first investigated the innate response of γδ T cells against ovarian tumor cell lines (A2780, A2780CR and OV4) using MTT cell viability and proliferation assay. Both cell lines showed a dose dependent decrease in proliferation after 24 h of co-culture (Fig. 2). The reduction in proliferation was prominent when ratio of γδ T cells and tumor cells was higher than 7.5∶1. When the ratio was increased to 30∶1, more than 60% reduction in proliferation was observed in both cell lines after 24 h of co-culture. After 48 h of co-culture, OV4 cell line showed a slightly higher reduction in cell proliferation compare to 24 h proliferation of the same cell line. However, there was no significant difference in the proliferation in A2780 cell line between 24 h and 48 h of co-culture with T cells. Similar results were obtained for A2780 CR cell line.

**Figure 3:**
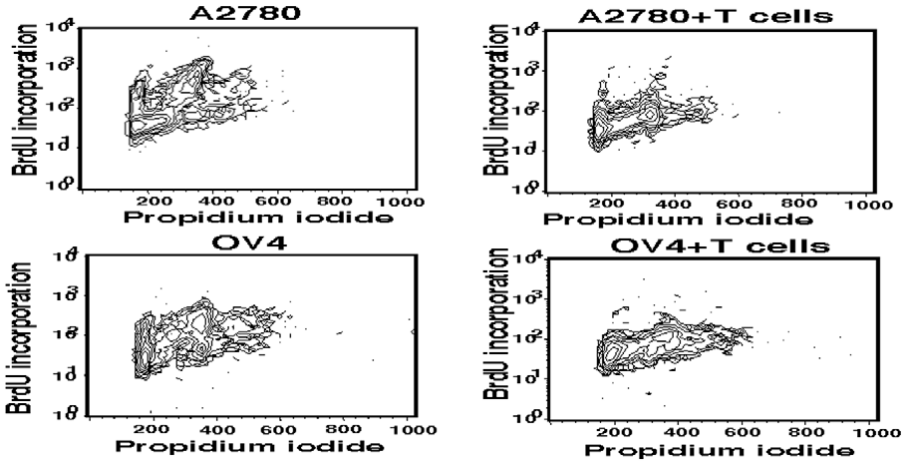
BrdU incorporation and propidium iodide (PI) co-staining in tumor cells. Ovarian tumor cells, A2780 and OV4 were co-cultured for 24 h in the presence or absence of γδ T cells at the ratio of 1∶15. After co-culture, cells were pulsed with BrdU for 5 hours and PI staining was performed prior to flowcytometric analyses.

We further explored whether the interaction of A2780 or OV4 cells with γδ T cells will influence on proliferation assessed by using BrdU and PI co-staining. Incorporation of BrdU into newly synthesized DNA permits detection of proliferating cells. Increased BrdU incorporation is indication of actively proliferating cells. Counterstain using PI can further allow resolution in G1, S, G2/M phase. Following exposure to γδ T cells, the A2780 cell line showed a reduced cell cycle progression in multiple phases (Fig. 3 and Fig. S3). This result was consistent with the reduction of cell cycle related protein expression in this cell line (discussed below). Similar profile was also observed in OV4 cell line. However, the down-regulation of cell cycle protein expression is not as significant as in A2780 cell line, which may indicate a different mechanism is involved. The most significant observation is that the inhibition of cell cycle progression is not restricted to a single phase. It seems that γδ T cells were able to influence the progression of ovarian tumor cells in multiple phases and this inhibition of progression seems to be common and share similar pattern in different ovarian cell lines.

### Signaling Pathway Lead to Inhibition of Tumor Cell Proliferation by γδ T Cells

**Figure 4:**
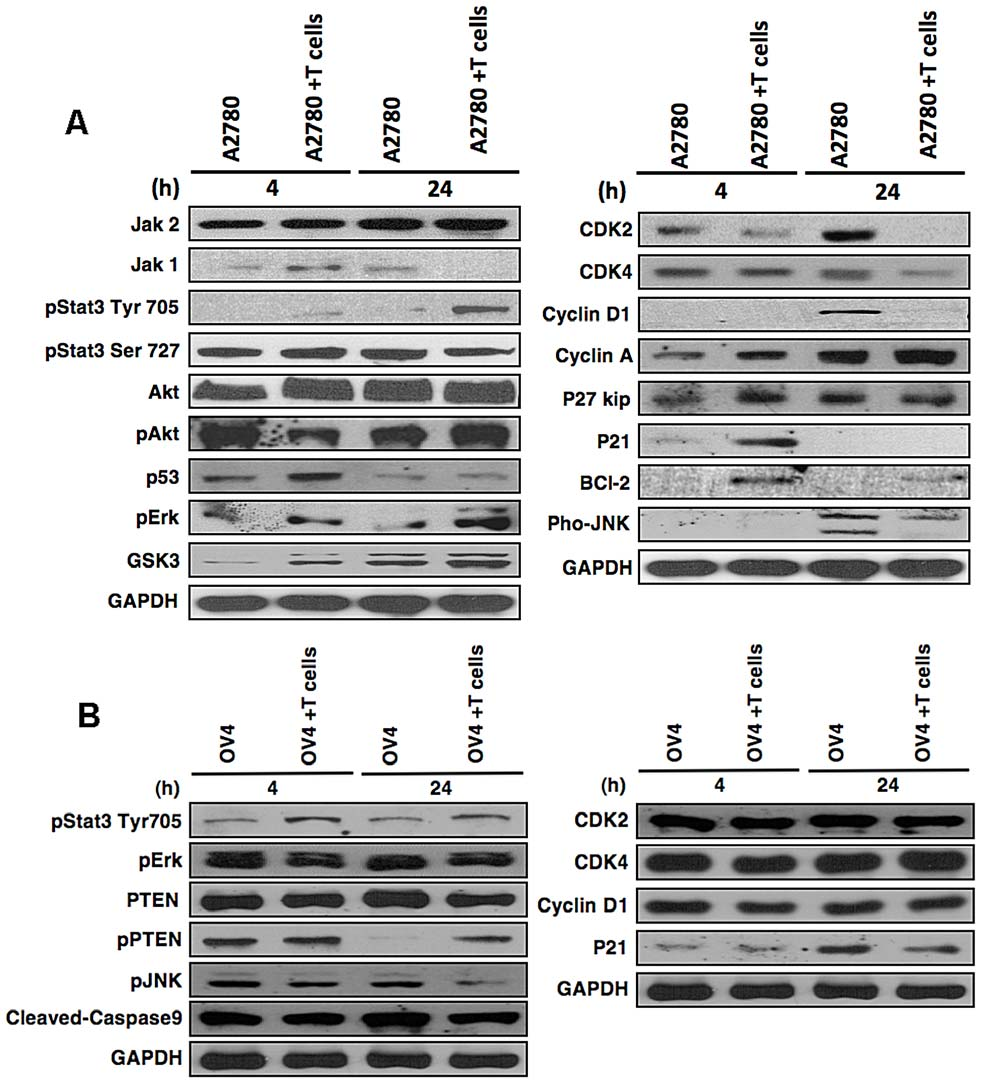
Expression of signaling molecules on tumor cells. Ovarian tumor cell lines were co-culturing with γδ T cells for 4 hours and 24 hours at 1∶5 ratio. After gentle removal of γδ T cells proteins were harvested from tumor cells and Western blot was performed for various molecules stated in A2780 (**A**) and OV4 (**B**) cells.

We analyzed the signaling molecules that are related to cell proliferation in both cell lines (Fig. 4). In A2780, the early up-regulation of p21^Waf1/Cip1^ indicated the activation p53/p21^Waf1/Cip1^ pathway, which further leads to the down-regulation of various cell cycle related proteins like cyclinD1, CDK2, and CDK4 in A2780 cell line (Fig. 4A, right panel). This results in cell cycle arrest, which is consistent with the reported results [23]. Some of the cell cycle related proteins such as cyclin D1 were found down-regulated after 48 h of co-culture indicating a long-term continuous interaction. GSK3 was found up-regulated, which s consistent with its role in regulating cyclin D1 proteolysis and sub-cellular localization. However, in OV4 cell line, a different pattern of protein expression was observed after co-culture with γδ T cells (Fig. 4B). The cell cycle related protein such as CDK4, CDK2 and cyclin D1 didn't show any significant change compare to A2780 cells, which suggest that the inhibition of proliferation in OV4 cells undergo a different pathway.

### Cell-to-Cell Interaction is Critical for γδ T Cells and Tumor Cell Communication

As two ovarian tumor cell lines showed different survival behavior after co-culture with γδ T cells, we tested whether cell-cell interaction between tumor cells and γδ T cells is important for cell survival/death. After transfer of the co-cultured media (supernatants from co-culture of tumor cells and γδ T cells) to the tumor cells, apparently we didn't see any differences in cell survival/death between the tumor cells cultured in conditioned media and normal RPMI/DMEM media (Fig. S2). These results indicated that cell-cell contact between the γδ T cells and tumor cells is important for the γδ T cell-mediated effect on tumor cells. Secretory factors did not play a significant role in limiting tumor proliferation or inducting apoptosis, however, might play a secondary role in this interaction.

### Tumor Cells Limit Surface Expression of MICA upon Exposure to γδ T Cells

**Figure 5:**
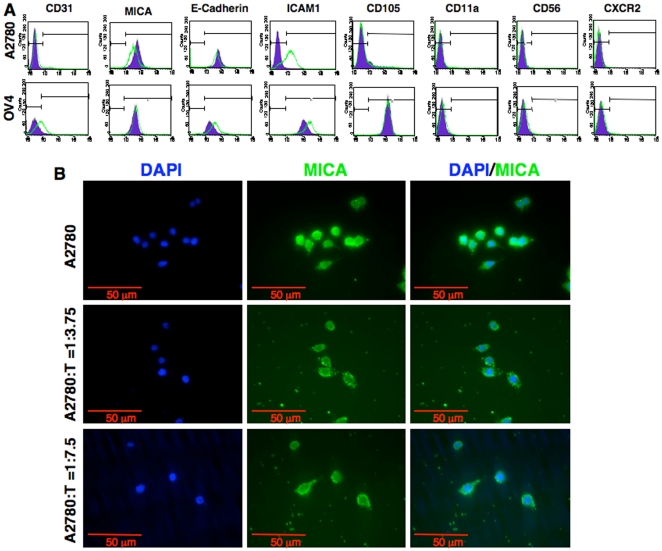
Expression of cell surface molecules on ovarian tumor cells after co-culture with γδ T cells. **A**. Flowcytometric analysis of various surface markers on tumor cells after co-culture with γδ T cells (1∶7.5 ratio) for 24 h. **B**. Expression of MICA on A2780 cells was evaluated by using immunocytochemical staining after co-culture with various concentration of γδ T cells.

To assess the role of molecules expressed on the surface of tumor cells for recognition of γδ T cells, we analyzed some of the relevant surface markers on tumor cells after co-culture with γδ T cells (Fig. 5A). We and others have reported previously that engagement of MICA/B with NKG2D present on Vγ2Vδ2 T cells resulted in a substantial enhancement of TCR-dependent T cell response to nonpeptide antigens and protein superantigens alike in various pathogenic infections and gliomas [19], [24]. We further explored whether the surface expression level of MICA/B played any role in co-stimulation of one-tumor cells better than other by γδ T cells. Flowcytometric analysis showed that both A2780 and OV4 cell lines express MICA/B on their surface. When co-cultured with γδ T cells the expression level of MICA/B did not change in OV4 cell line after 24 h. However, the surface expression of MICA/B was significantly down-regulated in A2780 cell line at the same time point, and this down-regulation could be dose dependent on γδ T cell: tumor cell ratio (Fig. 5AB). At the same time the surface expression levels of NKG2D was down regulated in γδ T cells when either of the tumor cell lines (Fig. S4). The expression level of CD31, a surface molecule with adhesion neovascularization and invasive functions in epithelial cells [25] were not changed in any of the cell lines tested. In both cell lines expression level of CD54 (ICAM-1) was up-regulated, which is consistent with cytokines effect on tumor cells [26]. However, differential expression of E-cadherin was observed. A2780 cell line showed no significant change of E-cadherin expression level, however, the expression level on OV4 was up-regulated minimally after 24 h of co-culture with γδ T cells. This may indicate reduced aggressiveness of the tumor cells, since up-regulation of E-cadherin is correlated with decreased invasive potential in ovarian carcinoma cells [27]. Both cell lines were negative for CD11a, CD56 and CXCR2 expressions. The CD105 expression level on OV4 was much higher than A2780 cells. It is not clear whether higher expression levels play any role in cytotoxic effect of γδ T cells, even though CD105 is part of the TGF-β receptor complex [28].

### Survival Signaling Molecules in Tumor Cells after Exposure to γδ T Cells

A number of tumor cell survival proteins, such as phospho-STAT3 Tyr705, phospho-Erk and total Jak1 were up-regulated in A2780 cells after exposure to γδ T cells (Fig. 4A, left panel). Other cell survival protein phospho-Akt, even though initially down-regulated, was remarkably up-regulated at 24 h time point, which might be responsible for inhibition of apoptosis [29]. Kinase protein of up-stream activation of stat3, Jak1 not the Jak2 was up-regulated as early as 4 h, which lead to an early response of Stat3. The up-regulation of pStat3Tyr705 is essential for gp130-mediated cell survival [30]. Even though Jak1 was down-regulated at 24 h, pStat3 Tyr705 remained in high level after 24 h of co-culture. PhosphoStat3Ser727 was not up-regulated in compare to pStat3Tyr705. The up-regulation of phosphor-Erk was observed as early as 4 h, is likely induced by p53, which was also up-regulated at 4 h. The up-regulation of p53 is an indicator of mitogen-activated protein kinase (MAPK) cascade under cellular stress condition [31]. Even though p53 was up-regulated at 4 h, the expression of p53 returned to normalcy after 24 h of co-culture, indicates that at the early time point tumor cell did experience with insult from γδ T cells. It was reported that an increase in the activation of Erk1/2 might lead to an up-regulation in the levels of a cyclin-dependent kinase (CDK) inhibitor, p21^Waf1/Cip1^, in human pancreatic adenocarcinoma cell lines [32], which is consistent with our findings that p21^Waf1/Cip1^ is up-regulated in this cell after co-culture with γδ T cells. However, for OV4 cell lines both activation and survival related proteins were down-regulation. Rather than up-regulation of pErk, the down-regulation of pErk was observed in OV4 cell line, which was consistent with the higher level of apoptosis (Fig. 4AB) cells. The levels of ADAMs and MMPs were also evaluated in A2780 and OV4 cell lines after co-culture with γδ T cells. However, the expression levels of all molecules tested were remarkably lower in A2780 cells compared to OV4 cells except ADAM17 (TACE), where no significant difference in expression level was observed (Fig. S5).

### Erk Signaling is Associated with Surface Expression of MICA on Tumor Cells

**Figure 6:**
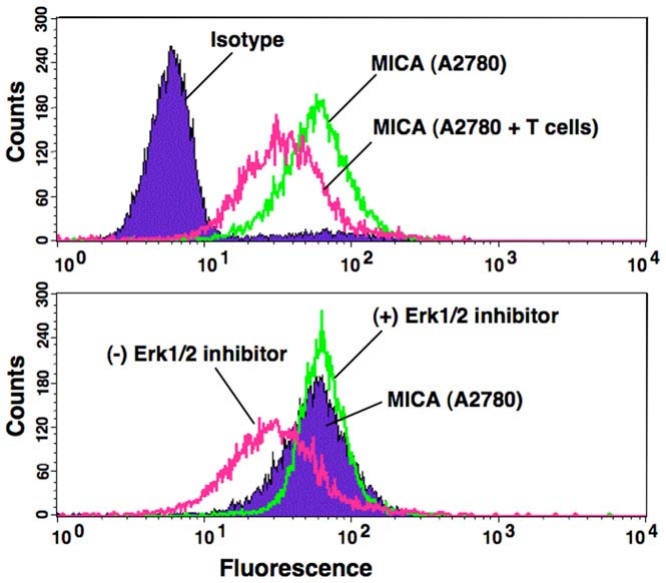
Surface expression of MICA on A2780 cells after co-culture with γδ T cells or Erk1/2 inhibitor. A2780 cells were co-cultured with γδ T cells (1∶7.5 ratio) for 36 h and flowcytometric analysis was performed to determine the level of surface expression of MICA on 2780 cells (upper panel). A2780 cells were co-cultured with γδ T cells for 24 h and gently removed the γδ T cells. A2780 cells were then cultured for another 12 h in presence or absence of Erk1/2 inhibitor. Flowcytometric analysis was performed for MICA expression (lower panel).

To further address the relationship between Erk signaling and the down-regulation of MICA/B in tumor cell surface, Erk pathway was blocked by using Erk inhibitor U0126. We found that level of pErk was increased in the resistant cell line, A2780 (where down regulation of MICA/B was observed) and no change in levels were observed in OV4 cell line after co-culture with γδ T cells. After incubation with Erk inhibitor with A2780 cells, the MICA/B surface expression was completely recovered to normal level (Fig. 6, lower panel). It is possible that Erk may play a critical role in the expression of MICA/B and recognition of γδ T cells as a result. The changes in pErk expression levels after exposure to γδ T cells may lead to differential regulation of MICA expressions, which might result in various levels in apoptosis in various tumor cells.

### Erk Signaling is Associated with Apoptosis of Tumor Cells to γδ T Cells

**Figure 7:**
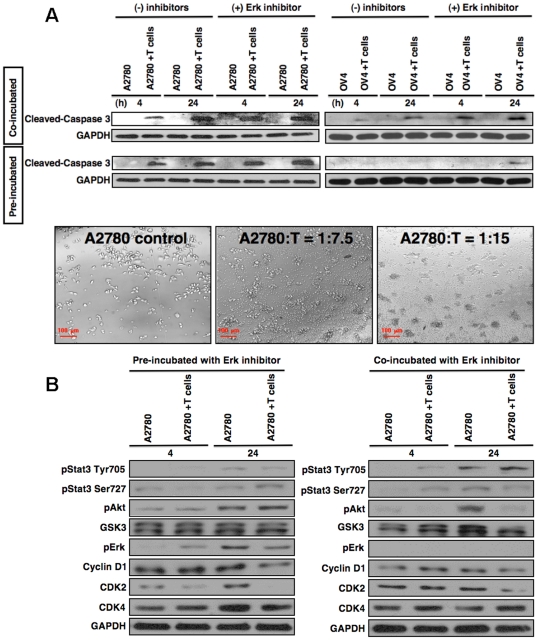
Effect of Erk1/2 inhibitor on tumor cell morphology and levels of signaling molecules after γδ T cells co-culture. **A.** A2780 or OV4 cells were pre-incubated with Erk inhibitor (−/+) for one hour or co-incubated with Erk inhibitors (−/+) until the termination of experiment. Cells were then co-cultured with γδ T cells (1∶5 ratio), and at various time points A2780 cells or OV4 cells were harvested after gentle removal of γδ T cells. Protein related to apoptosis (upper panel) was analyzed using Western blot. Cell morphology was determined under a phase contrast microscope after 1 hour of Erk-inhibitor pulse followed by 24 h of co-culture with γδ T cells at various ratios (lower panel). **B.** A2780 cells were pre-incubated with Erk inhibitor for one hour and then co-cultured with γδ T cells (1∶5 ratio) or co-incubated with Erk inhibitor during co-culture with γδ T cells and at various time points A2780 cells were harvested after gentle removal of γδ T cells. Proteins related to cell cycles and proliferations were analyzed using Western blot.

To study whether the resistance of A2780 cell line is related with up-regulation of pErk after exposure to γδ T cells, we designed two separate experiments using Erk-inhibitor. Tumor cells A2780 or OV4 were either pre-incubated or co-incubated with Erk-inhibitor during co-culture with γδ T cells. The level of cleaved caspase 3 expression was significantly up-regulated in both cases when co-cultured with γδ T cells at early time period (4 h), and remained high up to 24 h tested, compare to tumor cells without treatment (Fig. 7A, upper panels). These results indicated that inhibition of phosphor-Erk elevated the sensitivity to apoptosis of ovarian tumor cells to γδ T cells. This is also consistent with the morphological changes after co-culture with γδ T cells, which showed significantly higher amount of cluster formation, indicating greater cell death after pre-treatment with Erk-inhibitor (Fig. 7A, lower panel). Further protein analyses showed that the inhibition of pErk led to a change in the profile of pAkt and GSK3, which were found not significantly up-regulated after co-culture with γδ T cells compared to without treatment (Fig. 7B). However, the profiles of cell cycle related proteins, which were significantly down-regulated previously (Fig. 4A, right panel), when co-cultured with γδ T cells did not show marked decreased level (Fig. 7B).

## Discussion

Despite numerous efforts Vγ2Vδ2 T cell-mediated tumor immunity is yet to be completely understood. It is well established that human Vγ2Vδ2 T-cells are endowed with notable anti-tumor activity toward a large spectrum of malignant cells of diverse tissue origin [20] including lymphoma and myeloma cells [1]. Increasing evidence indicates that aminobisphosphonates, a γδ T cell antigen have a beneficial effect when used to minimize skeletal damage during tumor metastasis to bone [33]. Although aminobisphosphonates and bisphosphonates have long been known to inhibit osteoclastic activity by poorly defined mechanisms [34], the additional finding that aminobisphosphonates can activate Vγ2Vδ2 T cells *in vivo* and *in vitro* has raised the possibility that these compounds may be acting indirectly by enhancing the anti-tumor activity of Vγ2Vδ2 T cells [35]. Indeed, it was reported that when primed by bisphosphonates, Vγ2Vδ2 T cells could recognize a variety of nonpeptide antigens.

The stress-inducible molecule, MICA is widely expressed in many epithelial tumor cell lines. This ligand could be recognized by receptor NKG2D, the activatory C-type lectin, having a short cytoplasmic chain noncovalently associated with DAP10, and an adapter molecule that enables signal transduction, it has been identified on most γδT cells [36], [37]. However, the possible influence of the tumor cells in reduced cytotoxic effect of γδ T cells or immune evasion awaits more investigations. Herein we show that risedronate stimulation can induce an expansion of γδ T cells, especially Vγ2Vδ2 T cell subset, which confirms previous findings [20]. Vγ2Vδ2 T cells, after co-culture with A2780 and OV4 ovarian tumor cells, inhibit the proliferation of targeted tumor cells. Even though the cytotoxicity effect is reduced in A2780 cell line, the γδ T cells can still regulate tumor cell proliferation following a completely different pathway by modulating signaling molecules and inhibiting cell cycle related molecules of tumor cells. The proliferation inhibition of ovarian tumor cells seems to be more universal than expected. Various other tumor cell lines we have studied also showed reduction in cell cycle related protein (such as breast tumor cell lines, MZ01 and SkBR7; glioma cell lines LN229 and U87, unpublished data) other than A2780 and OV4 cells shown here. The effects of γδ T cells are much more complex, and can influence multiple phases of cell cycle, while drug treatment may target particular phase of the cell cycle to arrest tumor cell proliferation [38].

Human Vγ2Vδ2 T cells have a potent arsenal of effector functions at their disposal, including secretion of cytokines and chemokines, and cytotoxic activities that are likely to have antineoplastic effects. Cytotoxicity is accomplished through several pathways such as Fas- Fas ligand pathway and perforin and/or granulysin, molecules released from the effector cells that directly kills virus, bacteria or infected target cells [39]. Vγ2Vδ2 T cells bind to P-selectin and E-selectin, two adhesion molecules associated with extravasation of Th1 but not Th2 T cells into inflamed tissue [40]. These Vγ2Vδ2 T cells also respond to the C-C chemokines MCP-1, RANTES, MIP-1α and MIP-1β by transendothelial chemotaxis [41]. Thus, human Vγ2Vδ2 T cells have the characteristics of Th1 cells that possess multiple effector mechanisms capable of mediating anti-neoplastic effects. However, in our study the supernatant alone collected from co-culture of γδ T cells and tumor cells was not effective in either apoptosis or proliferation inhibition of tumor cells (Fig. S2). These findings indicate that the secretory molecules, if play any role, need a cell-cell contact to increase the local concentration and that might be important for the effectiveness for the limitation of tumor cell proliferation.

After cellular damage or under stress, cells have potential to arrest their cell cycle at various checkpoints temporarily for the repair of the damages. The two most important checkpoints are the G1/S and G2 checkpoints, where p53 play a significant role in maintaining checkpoints. Cyclin-dependent kinases (CDKs) are a family of protein kinases that regulate cell cycle in eukaryotes. CDKs are themselves regulated by CDK inhibitors (CKIs). Currently two main CKI families were discovered: INK4 family (p16^INK4A^, p15^NK4B^, p19^INK4D^ and p18^INK4C^) and the Cip/Kip family (p21^Waf1/Cip1^, p27^Kip1^ and p57^Kip2^). The INK4 family specifically inhibits CDK2 and CDK6 activities during the G1/S check point, while the Cip/Kip family inhibit CDK activity during all phases of the cell cycle [42]. The cytotoxic effect of γδ T cells on tumor cells could lead to apoptosis of tumor cells [20]. However, other interactions may also occur to tumor cells during exposure to γδ T cells. In tumor cells, activation of p53 has been reported to be able to induce cellular senescence. And the primary response of p53 activation is not apoptosis rather than cell cycle arrest [43]. Indeed, our study has shown that in A2780 cells, after the exposure to γδ T cells, experiencing series of events, which lead to the cell cycle arrest. These events may start as an up-regulation of p53, which lead to corresponding up-regulation of p21^Waf1/Cip1^ and down-regulation of cell cycle related protein such as cyclin D1, CDK2 and CDK4. The PI staining right after 24 hours of co-culturing tumor cells with γδ T cells didn't shown any phase specific arrest (Fig. S3). However, the BrdU and PI co-staining showed a significant slow-down in uptake of BrdU and inhibition of cell cycle progression at multiple phases. This is consistent with the multiple phase effects of CKI p21^Waf1/Cip1^ in cell cycle arrest rather than only G1/S phase. However, in A2780 cell line this cell cycle arrest also associated with up-regulation of surviving signaling proteins such as phospho-Erk, phospho-Akt and phospho stat3 Tyr705. The outcome of the complex signaling has resulted in a low level of apoptosis in A2780 cells. Indeed, by inhibiting Erk phosphorylation, A2780 cell line became more susceptible and showed higher level of cleaved-caspase 3 expression in early time point. PhosphoSTAT3 was found up-regulated during inhibition of of pErk this may be due to cross-talk of both pathways. The Erk signaling seems to be dominant here to promote the survival of ovarian tumor cells after exposure to γδ T cells [44], [45]. It was also reported that Erk activity induces cell cycle arrest at G2/M independent of p53, which may also contribute to the cell cycle arrest at multiple phases [46]. However, in OV4 cell line, even though the signaling profile is different, cell cycle arrest is also evident, which indicates that the inhibition of cell proliferation is common among different cell lines and different profile protein expression.

In summary, herein we report the mechanisms by which ovarian tumor cells escape immune recognition upon exposure to γδ T cells. Tumor cells when recognized by γδ T cells, cytotoxic pathway predominates and apoptotic signal is more effective as a result tumor cell become susceptible for lysis. However, some tumor cells can modulate their surface molecules as well as signaling molecules to hide recognition from immune cells and the pathway is dominant in the resistant ovarian tumor cells, A2780. Even though resistant tumor cells can escape immune recognition from γδ T cells by down regulating surface expression of MICA, γδ T cells can still be able to slow down the progression of the tumor cell proliferation by inhibiting cell cycle related molecules CDK2, CDK4 and Cyclin D1. Our findings clearly demonstrate that the effects of γδ T cells on ovarian tumor cells are more complicated than previous thought. By modulating certain signaling molecules, in this case, inhibiting Erk pathway, resistant ovarian tumor cells could be turned into susceptible towards γδ T cells-mediated immune recognition and resulted in lysis. As γδ T cells could be expanded in a large number using aminobisphosphonate *in vitro*, the possibility of expanding cells from peripheral blood and combining with molecules, which have potential for modulating particular signal might result in an effective immune therapy.

## Methods

### Derivation of γδ T Cells

Human peripheral blood was collected (30 ml) from adult healthy donors after obtaining the IRB approval from the Ohio State University Medical Center and obtaining written consents from donors. The ethic committee has also approved the procedure and records are saved in the laboratory logbook. Freshly collected blood was processed to isolate peripheral blood mononuclear cells (PBMC) following the similar protocol published earlier [19], [20], [47]. In brief, the peripheral blood was diluted twice with phosphate buffer saline (PBS, pH 7.4) and carefully layered over 10 ml of Ficoll-Paque Plus solution (GE Healthcare, Uppsala, Sweden). After 30 min of centrifugation in a swinging bucket rotor at 1400 rpm at room temp (24°C), the upper layer was aspirated out and the mononuclear cell layer (buffy coat) was collected. Buffy coat was washed three times with PBS to remove platelets. One million of PBMC in each well was stimulated with 10 µM risedronate in a 24-well plate using 1 ml RPMI 1640 supplemented with 10% fetal bovine serum (FBS, HyClone Lab Inc, Logan, UT), 2 mM glutamine, 1 nM β-mercapto ethanol, 1 nM HEPES and 100 IU of penicillin and streptomycin at 37°C incubator. Recombinant IL-2, 0.5 nM (PeproTech Inc. Rocky Hill, NJ) was added to the culture on days 3 and 7. Cells were split after day 10 using the complete RPMI 1640 media supplemented with 0.5 nM rIL-2. Flowcytometric (FACS) analysis was performed (using a FACS Calibur machine, BD Biosciences, CA) at day 14, to evaluate phenotype of the expanded cell. FACS analysis data revealed that 99.8% of the expanded cells were CD3+, and 89.5% of them were Vδ2+. Cells were used between 15–19 days of initial culture for further experiments discussed below.

### Morphology of Ovarian Tumor Cells after Co-Culture with γδ T Cells

Ten thousand ovarian tumor cells (A2780, OV4 and A2780CR; purchased from ATCC, VA, and used within six months after receipt) were cultured in a well of a 96-well culture plate in DMEM media supplemented with 10% FBS. The γδ T cells (T) were added to the well and co-cultured with ovarian cancer (C) cells with a ratio of C∶T = 1∶1 or 1∶7.5 or 1∶15 or 1∶30 for 24 h or 48 h at 37°C incubator with 5% CO_2_. Phase contrast micrographs were taken at 24 h and 48 h time points. The images were captured under an epifluorescence microscope (Axioplan2; Carl Zeiss) using Zeiss Axiovision imaging software. Separate sets of experiments were performed pre-treating A2780 cell line for one hour with Erk inhibitor U0126 at 10 µM final concentration or co-culturing with same concentration of U0126 for 24 h. Cells were then exposed to same ratio of γδ T cells following methods discussed above.

### Cell Viability and Proliferation Assays

Tumor cell proliferation was performed using MTT assays (3–4,5-Dimethylthiazol-2-yl-2,5-diphenyltetrazolium bromide), a yellow tetrazole, is reduced to purple formazan in living cells. The principal of the method uses the conversion of MTT to formazan via mitochondrial oxidation and colorimetric assay was used to detect the formazan as previously described with simple modifications [48]. Briefly, ten thousand of ovarian tumor cells (either OV4 or A2780WT or A2780CR) were co-cultured with γδ T cells at a ratio of 1∶30 or 1∶15 or 1∶7.5 or 1∶3.75 or 1∶1.9 or 1∶0.94 or 1∶0.47 or 1∶0, in a well of a 96-well plate and MTT assay was performed at 24 h or 48 h time points. Before performing MTT assays, γδ T cells were gently washed out once with 150 µl PBS to minimize the interference with tumor cells read-out. Most of the γδ T cells were washed away and very few tumor cells were drifted away during the process of washing observed under microscope. After washing out the γδ T cells, 100 µl of DMEM medium/well with a concentration of 500 µg/ml MTT reagent (Sigma) was added to the plate. Plates were incubated at 37°C in the incubator for 3 h and 100 µl of diluting solution (80% isopropyl alcohol, 10% HCl and 10% TritonX 100) was added to each well and mixed well by using auto mix shaker. The developed color value was recorded at 570 nM spectra and the reference spectrum was kept at 690 nM. MTT results were calculated using the equation:
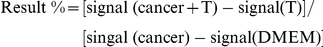



### Analysis of Apoptosis in Tumor Cells

To evaluate apoptosis tumor cells (150 K) were co-cultured with γδ T cells at 1∶10 ratio for 24 hours. All cells were harvested without any wash using non-enzymatic cell dissociation buffer. Cells were then incubated on ice with FITC-conjugated annexin V Ab for 30 min and washed with 1× PBS, and processed as described above. Flowcytometric analysis was performed to evaluate annexin V-positive cells. Cells negative for annexin V staining were considered as live cells, and annexin V-positive cells were considered as apoptotic cells.

### Effect of Conditional Media on Tumor Cell Proliferation

To verify whether cell-cell contact is necessary for γδ T cells to interact with tumor cells, ovarian tumor cells (A2780 or OV4, 10 K cells/well) were co-cultured with γδ T cells at a ratio of 1∶0, 1∶15 and 1∶30 in 96-well plates (in 200 µl volume). The γδ T cells were also cultured without tumor cells with a concentration of 150 K cells/well and 300 K cells/well in DMEM medium with 10% FBS for 24 h. A second set of similar tumor cells were grown at the same time without γδ T cells using similar plates. Tumor cells were cultured at the concentration of 60 K cells/well. After 24 h, 100 µl supernatant from first set was taken out and replaced with the medium of second set of tumor cells accordingly. MTT assay was performed after 24 h further incubation following the same procedure as described above.

### Flowcytometric Analysis

To assess the effect of γδT cells on surface molecules of tumor cells, γδ T cells were co-cultured with 300 K ovarian tumor cells per well either A2780 or OV4 at 7.5∶1 ratio in 6-well plate using RPMI medium (for A2780) or DMEM medium (for OV4) with 10% FBS. Cells were harvest using non-enzymatic cell dissociation solution (Sigma) after 24 h of co-culture and washed with PBS. Cells were then incubated with primary and secondary antibodies (if applicable) for 45 min each. Surface markers such as CD31, MICA, CD56, CD105, CD11a, CXCR2, ICAM-1 (PE-conjugated, Pharmingen), E-cadherin (Invitrogen, secondary antibody PE-conjugated) were tested using flowcytometry as described above.

To assess the effect of tumor cells towards the surface expression level of NKG2D on γδ T cells, similar experiments were performed as mentioned above, collected γδ T cells after incubation with tumor cells and flowcytometric analysis was performed using NKG2D Ab (PE-conjugated, Pharmingen).

### Immunocytochemical Study of MICA Expression on Tumor Cells

A2780 tumor cells were co-cultured with γδ T cells with various ratios such as 1∶0, 1∶3.75, and 1∶7.5 for 24 h. Cells were then fixed with 4% para formaldehyde at room temperature for 10 min, stained with anti-MICA Abs (kind gift from Dr. Spies, Seattle, WA) and DAPI. The staining was observed under an epifluorescence microscope (Axioplan2; Carl Zeiss) and images were captured with Zeiss Axiovision imaging software.

### BrdU Incorporation and Propidium Iodide Co-Staining

To evaluate the effect of γδT cells to arrest cell cycle of tumor cells, γδ T cells were co-cultured with three hundred thousand ovarian tumor cells A2780 or OV4 in a well at 15∶1 ratio in a 6-well plate. After 24 h, γδ T cells were removed by three times gentle washing with 1×PBS. Respective culture media such as DMEM (for OV4) or RPMI (for A2780) with 10% FBS and 10 µM BrdU were added to each plate. Cells were incubated at 37°C for another 5 h in an incubator. The cells were isolated by using non-enzymatic cell dissociation buffer (Sigma) and collected in 15 ml centrifuge tube, centrifuged at 300× g at 4°C. Cells were then washed with PBS once and fixed with 75% ethanol for over night. After centrifugation, pellet was resuspended in 2 M HCl solution and incubated for 20 min. Cells were washed with PBS and then resuspended with 0.1 M Sodium borate solution, and again incubated for 2 min to neutralize the residual acid. After washing with PBS, cells were incubated with primary antibody (anti-BrdU monoclonal antibody, Invitrogen) in 1× PBS containing 0.5% Tween-20 and 0.5% BSA for 45 min. Unbound primary antibody was removed using washing buffer (1×PBS containing 0.5% BSA). PE-conjugated secondary antibody (Pharmingen) was added and incubated with cells for another 45 min. Cells were then washed and incubated in PBS buffer containing RNase One™ Ribonuclease (10 U/ml, Promega) and propidium iodide (PI, 3 µM). Cells were then analyzed by using a flowcytometer (FACS Calibur, BD).

### Propidium Iodide Staining

The γδ T cells were co-cultured with three hundred thousand ovarian tumor cells, A2780 WT or OV4 in a well of a 6-well plate with a 10∶1 ratio. After 24 hours, γδ T cells were gently removed by washing with 1×PBS three times. Cells were dissociated and centrifuged at 300× g. Pellet was collected and washed with PBS once. Cells were then fixed and permeabilized with 80% ethanol over night. Fixed cells were centrifuged with and pellet was incubated in PBS for rehydration for 10 mins. Then cells were washed once with ice cold PBS. PI staining solution (Sigma, PI; 1∶1000 and RNase One; 1∶1000 in 1×PBS) was added to the cells and incubated for 20 mins at 37°C before analysis. PI was purchased from Sigma and BrdU antibody was from Pharmingen. Flowcytometry was performed using BD FACS Calibur™ machine and data analysis was performed by using Cell Quest software.

### Protein Analyses

Total protein analysis was performed using standard Western blot technology. Half a million of tumor cells (A2780 or OV4) were pre-seeded in 3 cm Petri dish for 10 h before adding γδ T cells. Control plates were also plated at the same time to maintain equal cell numbers, we did not observe any significant change on cell numbers after 10 h of pre-seeding. Fresh medium (RPMI for A2780 and DMEM for OV4) containing 10% FBS were replaced to the culture plates. Two and half million γδ T cells were added to the tumor cells to make a ratio of C∶T = 1∶5 (where ever applicable). Protein was isolated after 4 and 24 h of addition of γδ T cells. Before isolation of total protein, γδ T cells were gently removed by washing with 1× PBS. Various antibodies have been used for Western blot, such as total Jak1, pan Akt, pStat3 (Ser 705), pErk, CDK2, GAPDH, β-Actin (all from Cell Signaling Technology), p53, GSK3, CDK4, cyclin D1, cyclin A, p27, kip, p21^Waf1/Cip1^, cleaved-Caspase3, ADAM10, ADAM17, MMP9, MMP14 (all from Santa Cruz Biotech) to detect their level of expression on tumor cells at various time points of co-culture with γδ T cells.

To determine the role of Erk signaling in modulation of ovarian tumor cells (A2780 and OV4) and γδ T cells interaction, A2780 cells or OV4 cells (500 K cells plated a day before) were pre-treated with Erk inhibitor (U0126, 10 µM final concentration, Cell Signaling) for one hour and then co-cultured with γδ T cells (1∶5 ratio) for 4 h and 24 h. Western blot was performed from isolated proteins from tumor cells by removing γδ T cells following the same procedure as mentioned above. A separate set of experiments were performed by incubating A2780 or OV4 cells and γδ T cells (1∶5 ratio) together with Erk inhibitor (10 µM, U0126) for 4 h and 24 h, and then processed for Western blot analyses following above-mentioned protocol.

### Effect of Erk-Inhibitor in Recovery of Surface Expression of MICA

Ovarian tumor cells, A2780 (300 K) were co-cultured with γδ T cells with a ratios of 1∶7.5 or 1∶0 in a 6-well plate with RPMI medium containing 10% FBS. After 24 h of incubation, γδ T cells were gently removed by washing with PBS. FBS free RPMI medium was added to the plate with/without Erk inhibitor U0126 (10 µM, final concentration). Cells were cultured for another 12 h. Then cells were harvested and processed for flowcytometric analysis for surface expression of MICA.

### Statistical Analysis

Values were expressed as mean±SEM and statistical analysis was performed by ANOVA. Students t- test was also performed and the results were considered significant when values of p<0.05.

## Supporting Information

Figure S1 **Flowcytometric analyses of expanded γδ T cells.** Total human PBMC was stimulated with risedronate, an aminobisphosphonate in T-cell media supplemented with rIL-2 at day 3 and 7 and flowcytometric analysis was performed at day 17 for T cell subtypes after expansion.
(TIFF)

Figure S2 **Cell-to-cell contact is necessary not the cell secretory molecules to inhibit tumor cell proliferation.** A2780 or OV4 cells were co-cultured with γδ T cells and culture supernatants were added to the respective tumor cells to evaluate effects on proliferation of tumor cells using MTT assays. Tumor cell culture media or γδ T cell culture media were used as controls.
(TIFF)

Figure S3 **Cell cycle analysis of tumor cells using propidium iodide staining.** Tumor cells were co-cultured in presence or absence of γδ T cells at a ratio of 1∶10 for 24 hours. Propidium iodide staining was done after gentle removal of γδ T cells and flowcytometic analysis was performed for evaluation of cell cycle status.
(TIFF)

Figure S4 **Expression of NKG2D on γδ T cells after co-culture with tumor cells.** Filled histogram indicates surface expression level of NKG2D on γδ T cells without co-culture with any tumor cells. Green line indicates surface expression level of NKG2D on γδ T cells after co-cultured with tumor cell line A2780 at a ratio of 7.5∶1 for 24 hours. Magenta line indicate surface expression level of NKG2D on γδ T cells after co-cultured with tumor cell line OV4 at same ratio of cells and same time point.
(TIFF)

Figure S5 **The levels of ADAMs and MMPs in A2780 and OV4 cell lines after co-culture with γδT cells.** Ovarian tumor cell lines, A2780 or OV4 were co-cultured with γδT cells for 4 h and 24 h at 1∶5 ratio. After gentle removal of γδ T cells total proteins were harvested from tumor cells and Western blot was performed for levels of ADAMs and MMPs.
(TIFF)
