# Supplementary figures and images for: Human Ovarian Tumor Cells Escape γδ T Cell Recognition Partly by Down Regulating Surface Expression of MICA and Limiting Cell Cycle Related Molecules
Source: PLoS One. 2011 Sep 14;6(9):e23348. doi: 10.1371/journal.pone.0023348 (PMC3173356; doi:10.1371/journal.pone.0023348)

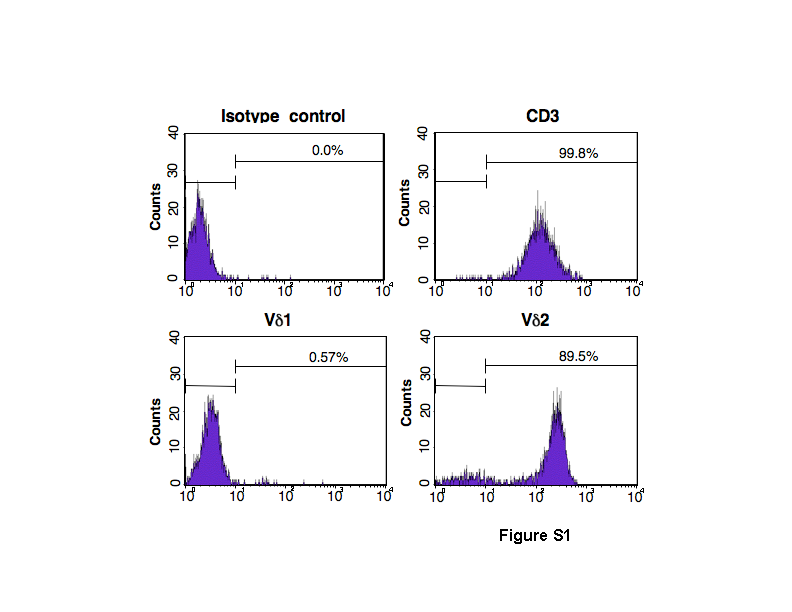

Supplement: Figure S1 — Flowcytometric analyses of expanded γδ T cells. Total human PBMC was stimulated with risedronate, an aminobisphosphonate in T-cell media supplemented with rIL-2 at day 3 and 7 and flowcytometric analysis was performed at day 17 for T cell subtypes after expansion. (TIFF) [file pone.0023348.s001.tiff]

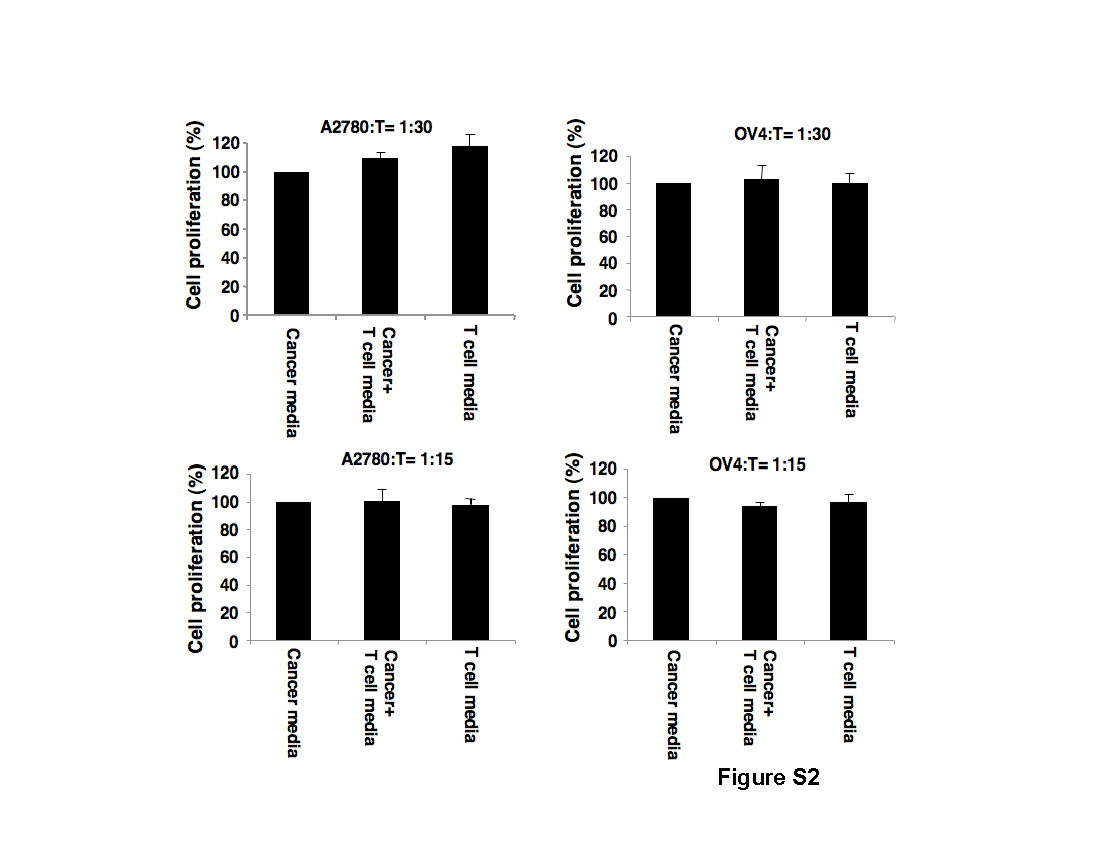

Supplement: Figure S2 — Cell-to-cell contact is necessary not the cell secretory molecules to inhibit tumor cell proliferation. A2780 or OV4 cells were co-cultured with γδ T cells and culture supernatants were added to the respective tumor cells to evaluate effects on proliferation of tumor cells using MTT assays. Tumor cell culture media or γδ T cell culture media were used as controls. (TIFF) [file pone.0023348.s002.tiff]

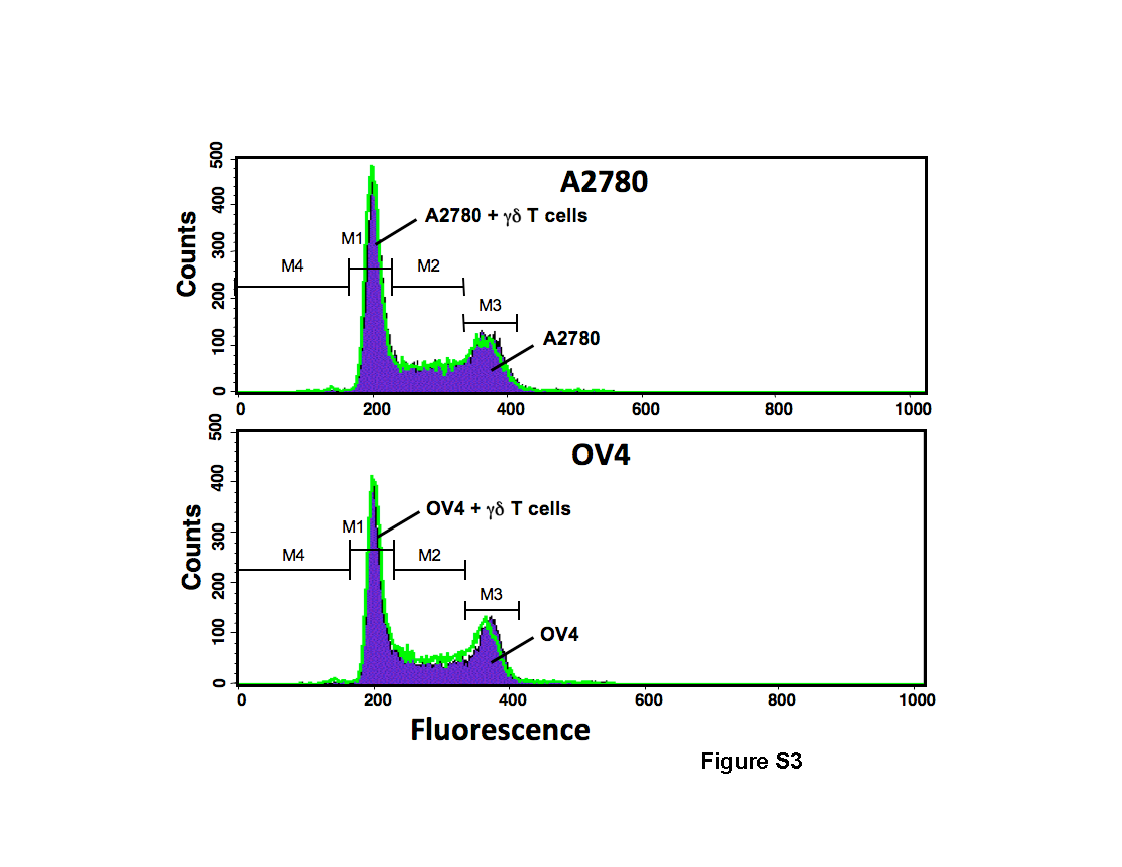

Supplement: Figure S3 — Cell cycle analysis of tumor cells using propidium iodide staining. Tumor cells were co-cultured in presence or absence of γδ T cells at a ratio of 1∶10 for 24 hours. Propidium iodide staining was done after gentle removal of γδ T cells and flowcytometic analysis was performed for evaluation of cell cycle status. (TIFF) [file pone.0023348.s003.tiff]

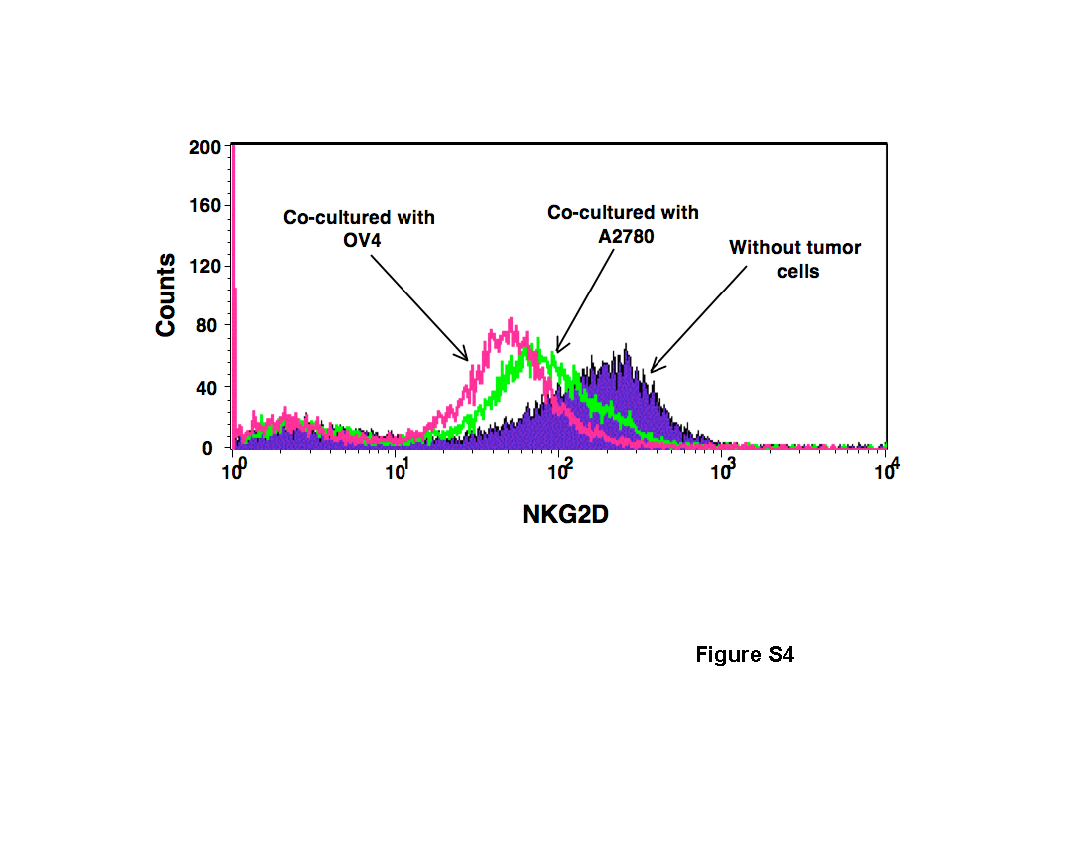

Supplement: Figure S4 — Expression of NKG2D on γδ T cells after co-culture with tumor cells. Filled histogram indicates surface expression level of NKG2D on γδ T cells without co-culture with any tumor cells. Green line indicates surface expression level of NKG2D on γδ T cells after co-cultured with tumor cell line A2780 at a ratio of 7.5∶1 for 24 hours. Magenta line indicate surface expression level of NKG2D on γδ T cells after co-cultured with tumor cell line OV4 at same ratio of cells and same time point. (TIFF) [file pone.0023348.s004.tiff]

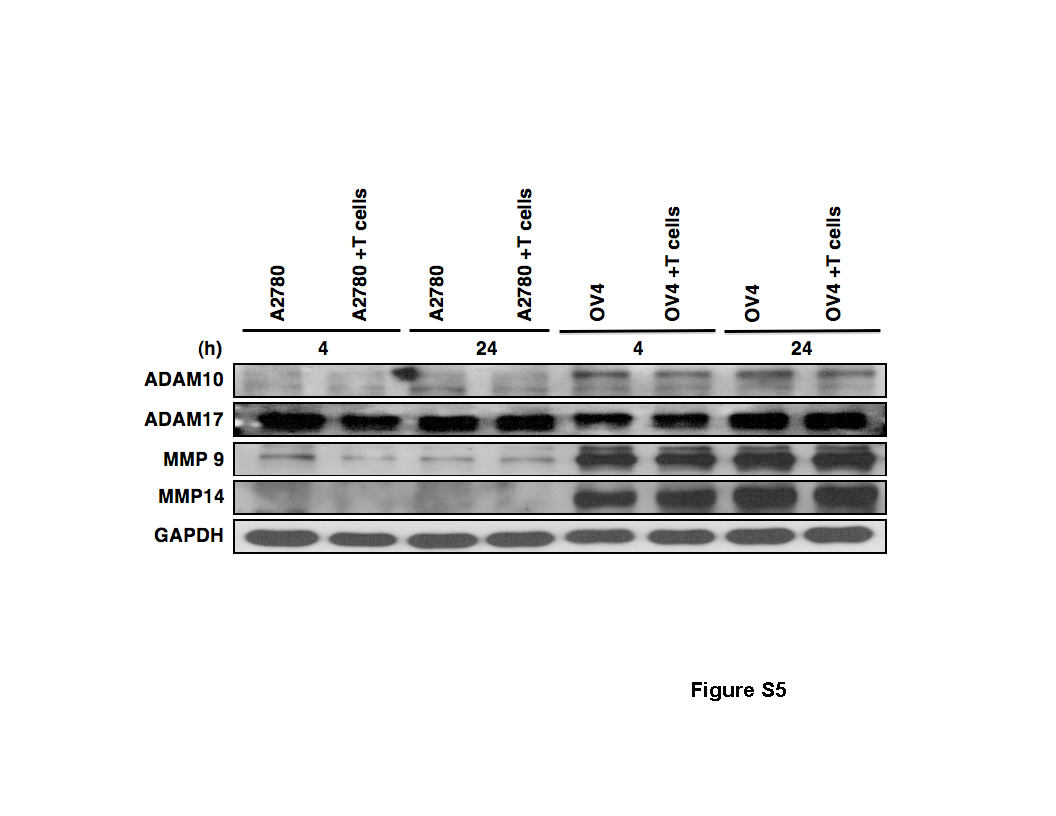

Supplement: Figure S5 — The levels of ADAMs and MMPs in A2780 and OV4 cell lines after co-culture with γδT cells. Ovarian tumor cell lines, A2780 or OV4 were co-cultured with γδT cells for 4 h and 24 h at 1∶5 ratio. After gentle removal of γδ T cells total proteins were harvested from tumor cells and Western blot was performed for levels of ADAMs and MMPs. (TIFF) [file pone.0023348.s005.tiff]
